# Slower Uncoating Is Associated with Impaired Replicative Capability of Simian-Tropic HIV-1

**DOI:** 10.1371/journal.pone.0072531

**Published:** 2013-08-13

**Authors:** Ken Kono, Eri Takeda, Hiromi Tsutsui, Ayumu Kuroishi, Amy E. Hulme, Thomas J. Hope, Emi E. Nakayama, Tatsuo Shioda

**Affiliations:** 1 Department of Viral Infections, Research Institute for Microbial Diseases, Osaka University, Suita, Osaka, Japan; 2 Department of Cell and Molecular Biology, Feinberg School of Medicine, Northwestern University, Chicago, Illinois, United States of America; The University of Hong Kong, Hong Kong

## Abstract

Human immunodeficiency virus type 1 (HIV-1) productively infects only humans and chimpanzees, but not Old World monkeys, such as rhesus and cynomolgus (CM) monkeys. To establish a monkey model of HIV-1/AIDS, several HIV-1 derivatives have been constructed. We previously generated a simian-tropic HIV-1 that replicates efficiently in CM cells. This virus encodes a capsid protein (CA) with SIVmac239-derived loops between α-helices 4 and 5 (L4/5) and between α-helices 6 and 7 (L6/7), along with the entire *vif* from SIVmac239 (NL-4/5S6/7SvifS). These SIVmac239-derived sequences were expected to protect the virus from HIV-1 restriction factors in monkey cells. However, the replicative capability of NL-4/5S6/7SvifS in human cells was severely impaired. By long-term cultivation of human CEM-SS cells infected with NL-4/5S6/7SvifS, we succeeded in partially rescuing the impaired replicative capability of the virus in human cells. This adapted virus encoded a G-to-E substitution at the 116^th^ position of the CA (NL-4/5SG116E6/7SvifS). In the work described here, we explored the mechanism by which the replicative capability of NL-4/5S6/7SvifS was impaired in human cells. Quantitative analysis (by real-time PCR) of viral DNA synthesis from infected cells revealed that NL-4/5S6/7SvifS had a major defect in nuclear entry. Mutations in CA are known to affect viral core stability and result in deleterious effects in HIV-1 infection; therefore, we measured the kinetics of uncoating of these viruses. The uncoating of NL-4/5S6/7SvifS was significantly slower than that of wild type HIV-1 (WT), whereas the uncoating of NL-4/5SG116E6/7SvifS was similar to that of WT. Our results suggested that the lower replicative capability of NL-4/5S6/7SvifS in human cells was, at least in part, due to the slower uncoating of this virus.

## Introduction

HIV-1 infection begins with the interaction and fusion of viral and cellular membranes. After fusion, a conical core, consisting of the two viral genomic RNAs and several viral proteins, is released into the cytoplasm of the target cell. The major component of the core is the viral capsid protein (CA). In the cytoplasm, CA eventually dissociates from the viral complex in a process termed uncoating. During this time, reverse transcription (RT) of the viral genomes occurs. The resultant double-stranded DNA associates with viral and cellular proteins, constituting the pre-integration complex (PIC). The PIC migrates into the nucleus, where the viral DNA integrates into the chromosomal DNA of the target cell.

HIV-1 uncoating was thought to occur immediately following viral fusion, as CA was undetectable in RT complexes isolated from infected cells [1–3]. Thus, CA was thought to have only a minor role in HIV-1 infection. However, subsequent reports indicated that mutations in CA decreased HIV-1 infectivity. Most of these CA mutant viruses displayed decreased levels of RT products [4–10]. On the other hand, the mutant virus Q63/67A, which encodes two Gln-to-Ala substitutions in CA, exhibited a defect in nuclear entry [4,11,12]. Changes in core stability caused by some of these CA mutations seem to affect uncoating kinetics, which may result in impaired RT or nuclear entry. Thus, timely uncoating was thought to be important for efficient HIV-1 infection. In agreement with this idea, anti-HIV factors TRIM5α and TRIMCyp were shown to bind viral core and accelerate uncoating, thus abrogating productive RT [13–17]. This observation suggests that the core persists as a defined structure for a certain period of time after fusion. Intriguingly, Yamashita et al. showed that CA is important for HIV-1 infection of non-dividing cells [11,18]. In addition, the transportin-SR2 (or TNPO3) -dependence of HIV-1 nuclear entry has been mapped to the HIV-1 CA [19,20]. These results also suggest a functional link between the HIV-1 CA and nuclear entry.

We previously generated simian-tropic HIV-1 that replicates efficiently in cynomolgus monkey (CM) cells [21]. This virus encodes a CA with SIVmac239-derived loops between α-helices 4 and 5 (L4/5) and between α-helices 6 and 7 (L6/7), along with the entire SIVmac239 *vif*. These SIVmac239-derived sequences allow HIV-1 to escape from restriction factors in monkey cells, including cyclophilin A (CypA), TRIM5α, and ApoB mRNA editing catalytic subunit (APOBEC) 3G. However, the replicative capability of this virus (NL-4/5S6/7SvifS) in human cells was severely impaired. By long-term cultivation of human CEM-SS cells infected with NL-4/5S6/7SvifS, we succeeded in partially rescuing the replicative capability of this virus in human cells [22]. This adapted virus encoded a G-to-E substitution at the 116^th^ position of the CA (NL-4/5SG116E6/7SvifS). Interestingly, this G116E mutation also occurred after adaptation in rhesus monkey cells [23].

In the work presented here, we examined the mechanism by which the replicative capability of NL-4/5S6/7SvifS was severely impaired in human cells.

## Materials and Methods

### Cells

The human kidney adherent 293T cells and the human cervical cancer HeLa cells were cultured in Dulbecco’s modified Eagle medium supplemented with 10% heat-inactivated fetal bovine serum (FBS). Cells of the human T cell line CEM-SS were maintained in RPMI 1640 medium supplemented with 10% FBS.

### Virus propagation

**Figure 1:**
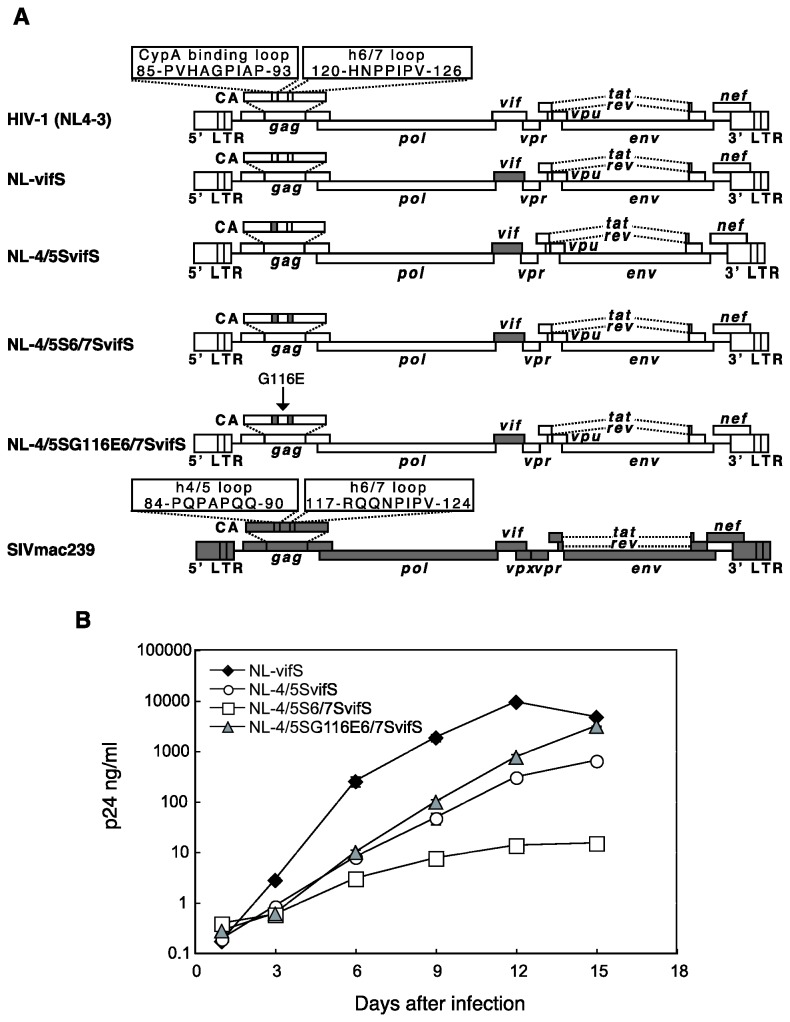
Structure of the simian-tropic HIV-1 clones and the replication properties in human cells. (A) White bars denote HIV-1 (NL4-3) and gray bars SIVmac239 sequences. (B) Equal amounts of NL-vifS (black diamonds), NL-4/5SvifS (white circles), NL-4/5S6/7SvifS (white squares), and NL-4/5SG116E6/7SvifS (gray triangles) were inoculated into human CEM-SS cells, and culture supernatants were collected periodically. p24 antigen levels were measured by ELISA. Error bars reflect actual fluctuations of duplicate infections.

Virus stocks were prepared by transfection of 293T cells with HIV-1 derivatives described previously [21,22,24] using polyethylenimine (PEI) (molecular weight, 25,000; Polysciences). As shown in Figure 1A, NL-vifS possesses the entire *vif* of SIVmac239 in the background of HIV-1 NL4-3 (NL-SVR in reference [24]). NL-4/5S6/7SvifS encodes CA with the SIVmac239-derived L4/5 and L6/7 in the background of NL-vifS [21]. NL-4/5SG116E6/7SvifS encodes a CA with an additional G-to-E substitution at the 116^th^ position, in the background of NL-4/5S6/7SvifS [22]. NL-Nh is a mutant of the NL4-3 proviral clone in which an *Nhe*I restriction enzyme cleavage site was blunted and re-ligated, introducing frame-shift mutations in the *env* gene [25]. For NL-Nh, GFP-expressing NL4-3-derived HIV-1 proviral clone MSMnG [25], and luciferase-expressing NL4-3-Luc-R-E- (NIH AIDS Research and Reference Reagent Program), the *BssH*II to *Apa*I fragment (corresponding to the majority of the *gag* gene) was replaced with the corresponding fragment of NL-4/5S6/7SvifS or NL-4/5SG116E6/7SvifS. Viral titers were measured with the RETROtek antigen ELISA kit (ZeptoMetrix, Buffalo, NY).

### Viral infections

CEM-SS cells (2 x 10^5^ per reaction) were infected with HIV-1 derivatives at titers equivalent to 20 ng of p24 per reaction. Culture supernatants were collected periodically, and p24 levels were measured using an ELISA kit.

### Real-time PCR analysis

CEM-SS cells (1 x 10^6^ per reaction) were infected with DNase I-pretreated HIV-1 derivatives at titers equivalent to 80 ng of p24 per reaction. DNase I pretreatment consisted of incubation with DNase I (20 units/ml in 10 mM MgCl_2_) for 30 min at room temperature. After 2 hr on ice, infected cells were washed with PBS, resuspended in medium, and returned to 37° C until harvesting at the indicated time point post-infection. Genomic DNA was extracted by using the QIAamp DNA Blood Mini kit (Qiagen). After digestion with 1 unit/μl *Dpn*I for 4 hr at 37° C, 30 ng of DNA was analyzed for U5/gag, 2-LTR, and Alu-HIV by real-time PCR using published primers and TaqMan probes [26,27] in an Applied Biosystems 7500 Real-Time PCR System.

### *In situ* uncoating assay

The *in situ* uncoating assay was conducted as previously described [11,28]. Briefly, the labeled virus was generated by cotransfecting 9 µg NL-Nh CA mutant proviral plasmid, 4 µg S15-dTomato-expressing plasmid, 4 µg vesicular stomatitis virus G protein (VSV-G)-expressing plasmid, and 1 µg GFP-Vpr-expressing plasmid into 10-cm plates of 293T cells using PEI. HeLa cells were spinoculated with the labeled virus for 2 hr at 16° C in the presence or absence of bafilomycin A (BafA) (Sigma). Virus-containing supernatant then was removed and replaced with 37° *C* *medium* in the presence or absence of BafA, shifted to 37° C, and fixed with 3.7% formaldehyde (Polysciences) in 0.1M PIPES buffer (pH 6.8) at the indicated time point post-infection. The fixed HeLa cells were permeabilized with blocking solution (0.1 M PIPES [pH 6.8], 10% normal donkey serum [Jackson ImmunoResearch Laboratories], 0.01% Triton X-100, 0.01% NaN_3_) for 5 min at room temperature, stained with anti-p24 mAb AG3.0 (NIH AIDS Research and Reference Reagent Program) in blocking solution without Triton X-100 for 1 hr at room temperature for primary staining, and secondarily stained with labeled Cy5 donkey anti-mouse antibodies (Jackson ImmunoResearch Laboratories) for 30 min at room temperature. Images were collected and deconvolved with a Deltavision microscope and software (Applied Precision). Following deconvolution, images were blinded for identity to remove bias during counting. The number of GFP-positive virions was assessed at each time point, and each virion was individually inspected for punctate dTomato fluorescent signal and p24 Cy-5 signal.

### Statistical analysis

Differences in luciferase activities, amounts of late RT products, and uncoating kinetics were evaluated with unpaired t tests.

## Results

### The replicative capability of NL-4/5S6/7SvifS was impaired in human cells, while that of NL-4/5SG116E6/7SvifS was partially rescued by a single amino acid mutation in CA

Several HIV-1 derivatives have been constructed to establish a monkey model of HIV-1/AIDS (Figure 1A). NL-4/5SvifS could replicate in CM cells [24]. Introduction into NL-4/5SvifS of SIVmac239 L6/7, which is a determinant of HIV type 2 (HIV-2) CM TRIM5α sensitivity [29], improved viral growth in CM cells [21]. However, the replicative capability of the resultant virus (NL-4/5S6/7SvifS) in human cells was greatly attenuated. After long-term cultivation of human CEM-SS cells infected with NL-4/5S6/7SvifS, we succeeded in partially rescuing the impaired replicative capability of the virus [22]. This adapted virus (NL-4/5SG116E6/7SvifS) encoded a G-to-E substitution at the 116^th^ position of NL4-3 CA sequence. Figure 1B shows the replication of NL-vifS that possesses the entire *vif* of SIVmac in the background of HIV-1, NL-4/5SvifS, NL-4/5S6/7SvifS, and NL-4/5SG116E6/7SvifS in human CEM-SS cells. Consistent with our previous report [22], the replicative capability of NL-4/5S6/7SvifS was severely impaired in human cells (Figure 1B). On the other hand, the replicative capability of NL-4/5SG116E6/7SvifS was improved compared with that of NL-4/5S6/7SvifS, and slightly better than that of NL-4/5SvifS, even though replication did not reach the levels seen with NL-vifS.

**Figure 2:**
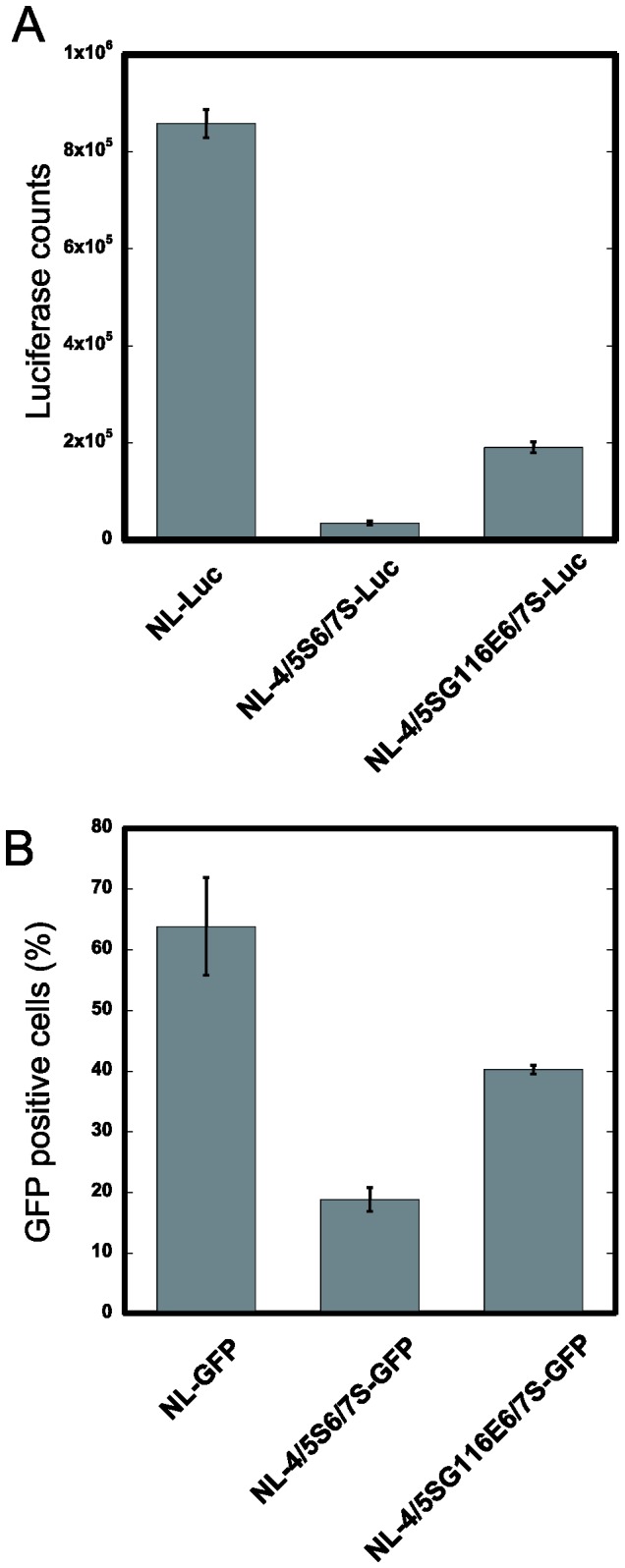
Single round infection assays. (A) 3X10^5^ CEM-SS cells were infected with viral titers equivalent to 5 ng of p24 of VSV-G-pseudotyped luciferase-expressing viruses with NL4-3 CA (NL-Luc), 4/5S6/7S CA (NL-4/5S6/7S-Luc), or 4/5SG116E6/7S CA (NL-4/5SG116E6/7S-Luc). The luciferase activity was measured at 48 hr after infection by a luminometer. Error bars reflect the SD of triplicate infections. Presented data are representative of two independent experiments using a different set of molecular clones. (B) 3X10^5^ CEM-SS cells were infected with viral titers equivalent to 80 ng of p24 of VSV-G-pseudotyped GFP-expressing viruses with NL4-3 CA (NL-GFP), NL-4/5S6/7S CA (NL-4/5S6/7S-GFP), or NL-4/5SG116E6/7S CA (NL-4/5SG116E6/7S-GFP). The GFP-positive cells were counted at 24 hr after infection by a flow cytometer. Error bars reflect the SD of triplicate infections. Presented data are representative of two independent experiments using a different set of molecular clones.

We then inoculated CEM-SS cells with VSV-G-pseudotyped luciferase- expressing HIV-1 vector encoding wild type (WT), 4/5S6/7S, or 4/5SG116E6/7S CA. As shown in Figure 2A, infectivity was significantly reduced by the 4/5S6/7S mutation (p<0.0001), and infectivity was restored by addition of the G116E mutation to 4/5S6/7S (p=0.0004). Similar results were obtained when we used a VSV-G-pseudotyped GFP-expressing version of the HIV-1 vector (Figure 2B). These results clearly indicated that the different replicative capability of the viruses was due mainly to effects at the early stage of viral replication.

### Levels of NL-4/5S6/7SvifS RT products were decreased at 12 hours after infection

**Figure 3:**
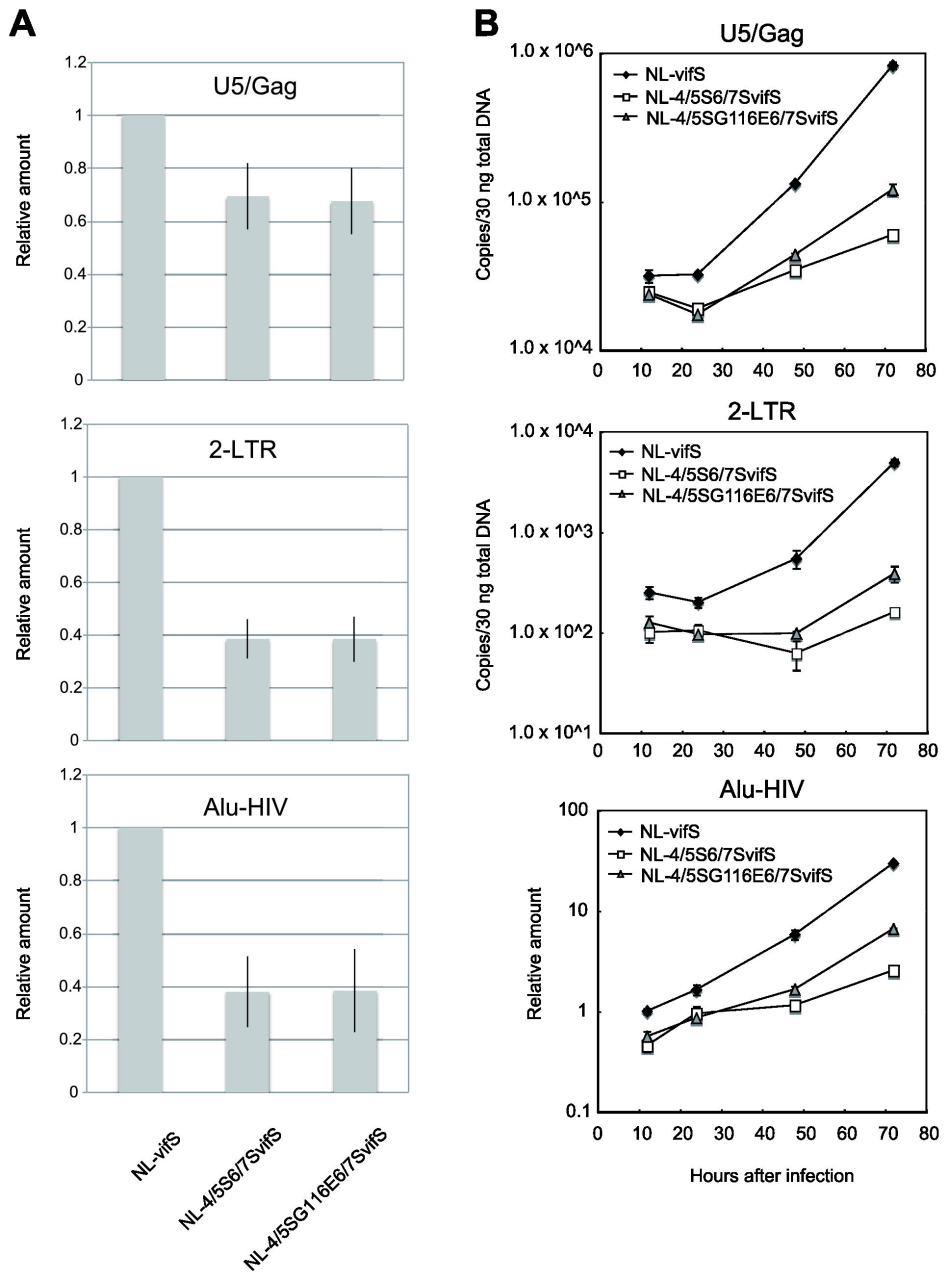
Measurement of the reverse transcribed products of simian-tropic HIV-1 in human cells. (A) CEM-SS cells were infected with NL-vifS, NL-4/5S6/7SvifS, or NL-4/5SG116E6/7SvifS, and DNA was extracted at 12 hr after infection and subjected to real-time PCR assays using U5/gag primers for late reverse transcription (RT), 2-LTR primers for nuclear transported viral DNA, and Alu-HIV primers for integrated DNA. Mean relative amounts of U5/gag, 2-LTR, and Alu-HIV products obtained from three independent experiments (the amount in the NL-vifS sample at 12 hr after infection is set at 1) are indicated. Mean numbers of U5/gag, 2-LTR, and Alu-HIV copies per 30 ng of total DNA of NL-vifS-infected cells were 39695, 187, and 2.17, respectively. Error bars reflect the SD of the three independent experiments. (B) CEM-SS cells were infected with NL-vifS, NL-4/5S6/7SvifS, or NL-4/5SG116E6/7SvifS, and DNA was extracted at 12, 24, 48, and 72 hr after infection and subjected to real-time PCR assays as described above. The number of viral DNA (U5/gag and 2-LTR) copies per 30 ng of total DNA and relative amount of Alu-HIV products (the amount in the NL-vifS sample at 12 hr after infection is set at 1) is indicated. Error bars reflect the SD of triplicate values of real-time PCR. Presented data are representative of three independent experiments.

To determine which step of NL-4/5S6/7SvifS early infection stage was impaired, we first measured RT products of replication-competent viruses NL-vifS, NL-4/5S6/7SvifS, and NL-4/5SG116E6/7SvifS in CEM-SS cells. At 12 hr after infection, the amounts of U5/gag (late RT products) and 2-LTR circles (a surrogate for nuclear entry) of NL-4/5S6/7SvifS were 69.4% (p=0.0270) and 38.6% (p=0.0003) of those of NL-vifS, respectively (Figure 3A). These results suggested that NL-4/5S6/7SvifS has defects in both RT and nuclear entry. On the other hand, the amount of Alu-HIV (integrated viral DNA) of NL-4/5S6/7SvifS was 38.2% (p=0.0029) that of NL-vifS, being comparable to that of 2-LTR circles. These results suggested that NL-4/5S6/7SvifS has WT-like ability to integrate after nuclear entry. In the case of NL-4/5SG116E6/7SvifS, the amount of late RT, 2-LTR, and Alu-HIV were 67.5% (p=0.0216), 38.4% (p=0.0005), and 38.5% (p=0.0052) of NL-vifS, respectively. These results suggested that NL-4/5SG116E6/7SvifS also was impaired for RT and nuclear entry. We failed to detect any significant recovery of late RT (p=0.88), 2-LTR (p=0.98), or Alu-HIV (p=0.98) of NL-4/5SG116E6/7SvifS by addition of the G116E mutation to NL-4/5S6/7SvifS.

We then measured RT products during a 72-hr time course (Figure 3B). The amount of late RT products of NL-4/5S6/7SvifS and NL-4/5SG116E6/7SvifS were decreased to similar extents at 24 hr after infection (Figure 3B upper panel), likely because of degradation of unproductive products. Supporting this idea, the levels of Alu-HIV, an outcome of productive infection, continued to increase in cells infected with these viruses (Figure 3B lower panel). On the other hand, the level of late RT of NL-vifS at 24 hr after infection was almost the same as that at 12 hr after infection. This persistence is likely due to the balance between degradation of unproductive RT products from the first round of infection and newly generated RT products from the second-round infection by the progeny viruses, since this experiment used replication-competent viruses. In the cases of NL-4/5S6/7SvifS and NL-4/5SG116E6/7SvifS, the RT products from the second-round infection also would be impaired. Thus, these viruses were not expected to overcome the degradation of unproductive RT products of the initial infection. The difference of late RT, 2-LTR, and Alu-HIV between NL-4/5S6/7SvifS and NL-4/5SG116E6/7SvifS gradually expanded at 48 and 72 hr after infection, presumably due to the effects of multiple rounds of infection. This result was in good agreement with that of the p24 production shown in Figure 1B.

### The levels of late RT product of NL-4/5S6/7SvifS were increased at the earlier time points of infection

**Figure 4:**
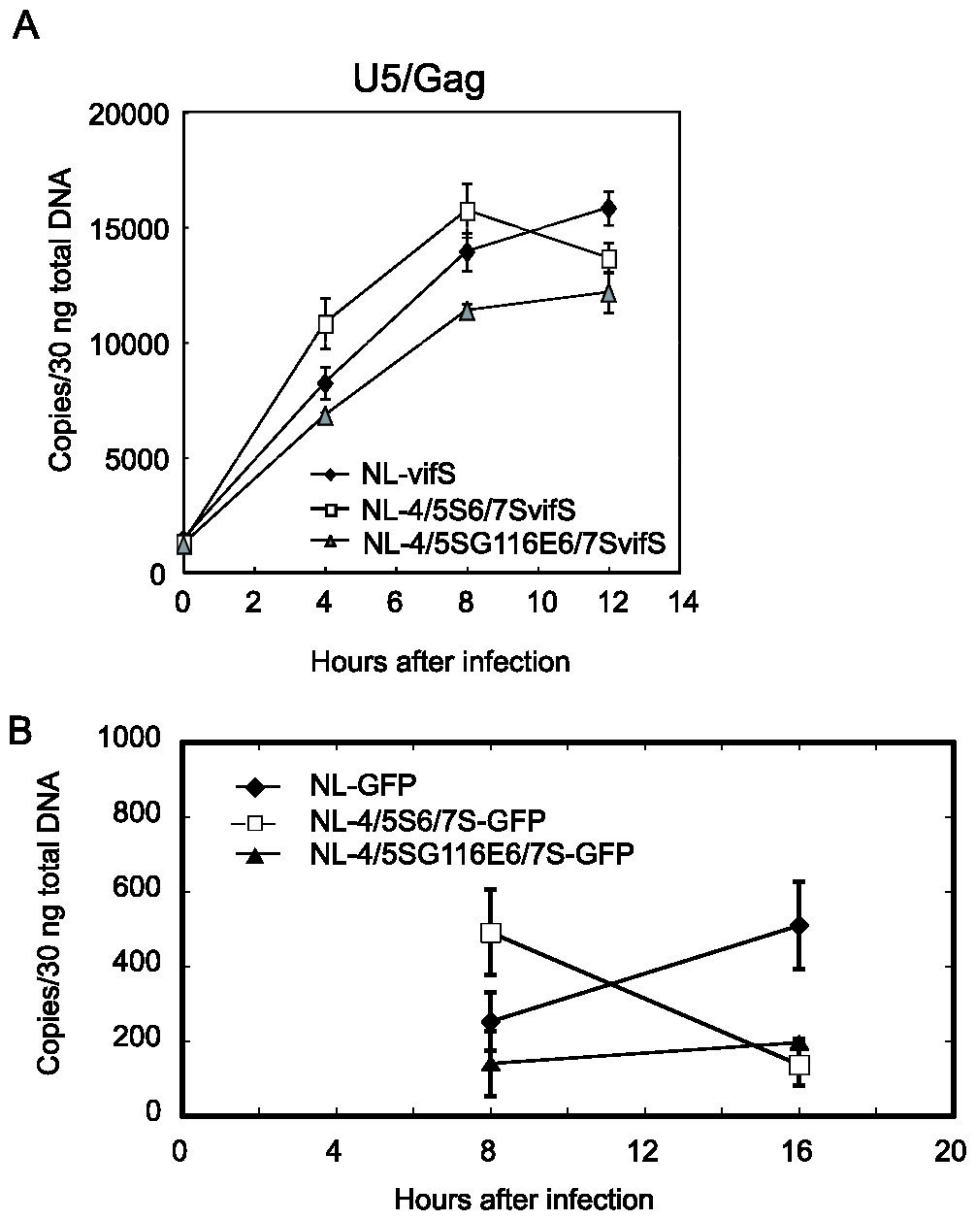
Measurement of the U5/gag (late RT products) during a 12-hr time course. (A) NL-vifS (black diamonds), NL-4/5S6/7SvifS (white squares), and NL-4/5SG116E6/7SvifS (gray triangles) were inoculated into human CEM-SS cells. Genomic DNA was extracted at the indicated time point post-infection and subjected to real-time PCR assays using U5/gag primers. The number of U5/gag copies per 30 ng of total DNA is indicated. Error bars reflect the SD of triplicate measurements of real-time PCR. Presented data are representative of two independent experiments. (B) VSV-G-pseudotyped GFP-expressing viruses with NL4-3 CA (NL-GFP, black diamonds), 4/5S6/7S CA (NL-4/5S6/7S-GFP, white squares), and 4/5SG116E6/7S CA (NL-4/5SG116E6/7S-GFP, black triangles) were inoculated into human CEM-SS cells. Real-time PCR assays using U5/gag primers were performed as described above. Error bars reflect the SD of triplicate infections. Presented data are representative of three independent experiments.

To determine the mechanisms of the decreased RT production of NL-4/5S6/7SvifS and NL-4/5SG116E6/7SvifS, we analyzed RT at earlier time points after infection. Contrary to our expectation, the amount of late RT products of NL-4/5S6/7SvifS exceeded that of NL-vifS at 4 and 8 hr after infection (Figure 4A). This result indicated that the kinetics of RT product generation was faster for NL-4/5S6/7SvifS than for NL-vifS, despite the fact that the 12-hr levels of late RT products were lower with NL-4/5S6/7SvifS than with NL-vifS (Figures 3A and 4A). The late RT production of NL-4/5S6/7SvifS peaked at 8 hr after infection before decreasing at 12 hr after infection. In contrast, late RT products of NL-vifS gradually increased until 12 hr after infection. The peak amount of late RT products with NL-4/5S6/7SvifS was comparable to that with NL-vifS. Thus we conclude that NL-4/5S6/7SvifS had a defect not in RT but in nuclear entry, and that the synthesized viral cDNA that failed to enter the nucleus was degraded.

In a sharp contrast to NL-4/5S6/7SvifS, NL-4/5SG116E6/7SvifS yielded reduced amounts of late RT products compared to NL-vifS at the respective time points. Similar to NL-vifS, however, the late RT products of NL-4/5SG116E6/7SvifS gradually increased until 12 hr after infection. These findings also were unexpected and indicated that the single G-to-E substitution (which at least partially rescued the impaired replicative capability of NL-4/5S6/7SvifS in human cells) also attenuated late RT at the earlier time points. Therefore, the mechanism underlying decreased late RT product levels of NL-4/5S6/7SvifS at 12 hr after infection seemed to be totally different from that of NL-4/5SG116E6/7SvifS.

Next, we measured amounts of RT product of aforementioned VSV-G-pseudotyped GFP-expressing HIV-1 vectors in a single-round infection assay to confirm the results seen with replication-competent viruses. The absolute copy numbers of RT products of GFP-expressing viruses were less than those of replication-competent viruses, probably due to increase of genome size by reporter gene insertion. However, amounts of the late RT products of the virus encoding 4/5S6/7S CA at 8 hr after infection exceeded those of the virus encoding NL4-3 CA (p=0.04, Figure 4B), as observed in replication-competent viruses. At 16 hr after infection, the amounts of the late RT products of the virus encoding 4/5S6/7S CA were less than those of the virus encoding NL4-3 CA (p=0.007, Figure 4B), consistent with the results of replication-competent viruses. Furthermore, amounts of late RT products of the virus encoding 4/5S116E6/7S CA were lower than those of the virus encoding NL4-3 CA at 16 hr after infection (p=0.009, Figure 4B). Thus, the results of replication-incompetent viruses clearly confirmed the results of replication-competent viruses. Similar results also were obtained when we used VSV-G-pseudotyped NL-Nh versions of the viruses (data not shown).

### The uncoating kinetics of NL-4/5S6/7S was slower than that of the virus with the NL4-3 CA

**Figure 5:**
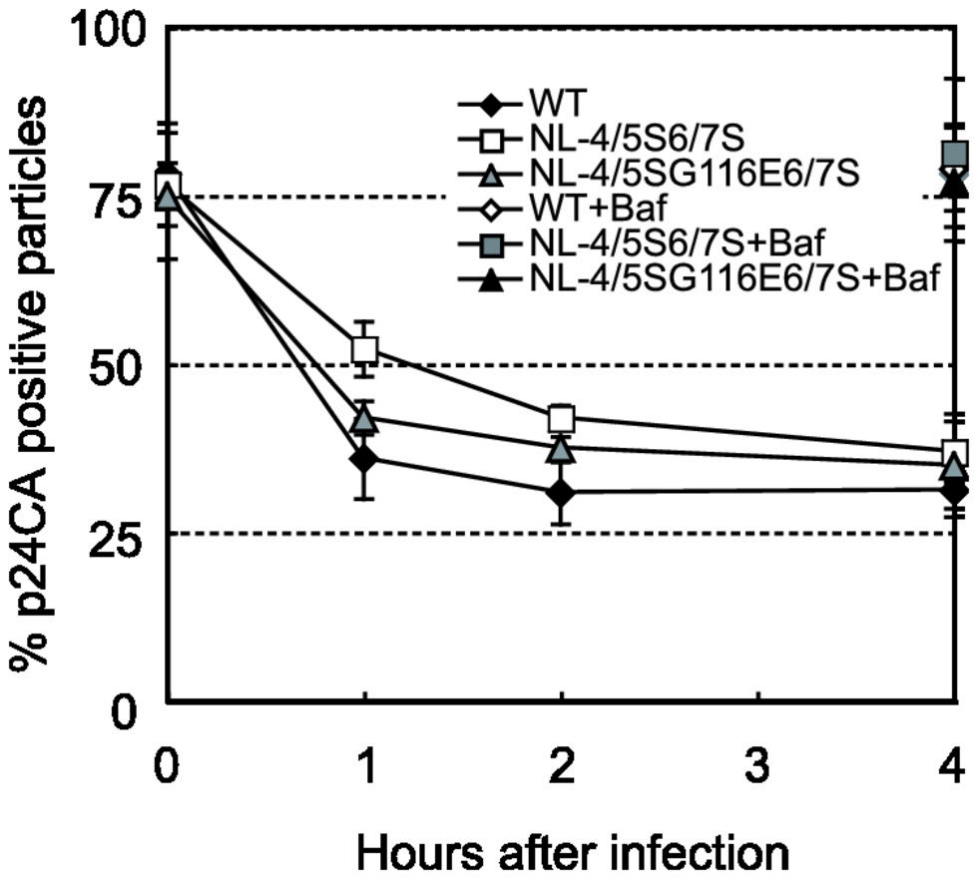
*In situ* uncoating assay. HeLa cells were spinoculated with VSV-G-pseudotyped, S15-dTomato, GFP-Vpr -labeled NL-Nh (WT; black diamonds), NL-Nh with 4/5S6/7S CA (NL-4/5S6/7S, white squares), or NL-Nh with 4/5SG116E6/7S CA (NL-4/5G116E6/7S, gray triangles) for 2 hr at 16° C in the presence or absence of bafilomycin A (BafA). Infection was synchronized by washing off inocula and replacing with 37° *C* *medium* . At the indicated time post-infection, the cells were fixed, immunostained for p24 CA (Cy-5), and imaged. The identity of the samples was blinded before counting. GFP-positive puncta then were quantified and individually examined for the presence of dTomato and Cy-5 (p24 CA) signals. The percentage of the total number of fused (dTomato-) virions that stained for p24 CA over time following fusion is shown. The 0-hr time point and BafA (+) samples represent total number of GFP-positive virions that stained positive for p24 CA. For BafA treatment, only data from the 4-hr time points on 4/5S6/7S CA (NL-4/5S6/7S+Baf, a grey square), or 4/5SG116E6/7S CA (NL-4/5G116E6/7S+Baf, a black triangle) are shown. The results shown are means and SD from three independent experiments. Actual numbers of counted dots are provided in Table S1.

Several studies have reported that mutations in CA affected viral core stability and resulted in deleterious effects on RT [4] or nuclear entry [12]. To determine whether the CA mutations in NL-4/5S6/7SvifS or NL-4/5SG116E6/7SvifS affect the core stability, we performed an *in situ* uncoating assay according to the method described previously [11,28]. For our experiment, a replication-incompetent virus (NL-Nh), which carries a frame-shift mutation in the *env* gene, was used as the wild type virus. The CA of NL-Nh was replaced with that of NL-4/5S6/7SvifS or NL-4/5SG116E6/7SvifS, since the virus had to be pseudotyped with VSV-G. NL-Nh, NL-Nh mutant encoding 4/5S6/7S CA, and NL-Nh mutant encoding 4/5SG116E6/7S CA were labeled with GFP-Vpr, while the viral membrane was labeled with S15-dTomato; the membrane label was expected to disappear after productive fusion of the virion into the cytoplasm. To provide a negative control reaction, bafilomycin A (BafA) was included to block fusion of the virus and cellular membranes. This control was used to confirm that unfused viral particles fail to undergo uncoating. Infection was synchronized and at various times after infection the cells were fixed and stained with an antibody to p24 CA. The total number of complexes that entered the cytoplasm (green spots that lost S15-dTomato) was counted, and the number of complexes that contained CA (coated) was compared to the number of complexes that lost CA staining (uncoated). The data was graphed at each time point as the % of fused CA-positive (coated) cytoplasmic particles (Figure 5). Actual numbers of counted dots are shown in Table S1. At 1 and 2 hr after infection, virus encoding 4/5S6/7S CA had a higher percentage of CA-positive particles than did the virus encoding NL4-3 CA; the difference was significant (p=0.018 and p=0.018 for 1 and 2 hr, respectively) at each time point. In comparison, virus encoding 4/5SG116E6/7S CA had amounts of CA-positive particles that were not significantly different from those seen with the virus encoding NL4-3 CA (p=0.18 and p=0.08 for 1 and 2 hr, respectively). The differences between 4/5S6/7S and 4/5SG116E6/7S viruses at 1 and 2 hr after infection were small but statistically significant (p=0.021 and p=0.037 respectively). These results suggested that the uncoating kinetics of NL-4/5S6/7SvifS was slower than that of NL-vifS, while the uncoating kinetics of NL-4/5SG116E6/7SvifS was similar to that of NL-vifS.

## Discussion

We previously constructed a simian-tropic HIV-1 NL-4/5S6/7SvifS that can replicate well in CM cells [21,22]. However, the replicative capability of this virus in human cells was severely impaired. NL-4/5S6/7SvifS showed nearly normal levels of Gag processing and human TRIM5α sensitivity similar to that of NL4-3 [22]. In the present study, we showed that the amount of RT products of NL-4/5S6/7SvifS was reduced compared to those of NL-vifS at 12 hr after infection. Surprisingly, however, the amount of the RT products of NL-4/5S6/7SvifS at 4 and 8 hr after infection was elevated compared to that of WT. Analysis of 2-LTR and integrated HIV DNA suggested that NL-4/5S6/7SvifS had a defect in nuclear entry but not in integration. By contrast, NL-4/5SG116E6/7SvifS, which encodes a single G116E substitution in CA, showed partial restoration of replicative capability, even though the amount of the RT products was apparently reduced. These results indicated that the G-to-E substitution at the 116^th^ position of CA impaired RT production but restored the defect of NL-4/5S6/7SvifS in the subsequent step.

Mutations in CA have been reported to affect viral core stability, resulting in deleterious effects on RT [4,10] or nuclear entry [12]. In the work described here, VSV-G-pseudotyped virus with NL-4/5S6/7S CA showed slower uncoating kinetics. Thus, it is possible that the hyper-stable core of NL-4/5S6/7SvifS affects nuclear entry, resulting in lower replicative capability in human cells. It remains unclear why the hyper-stable core would be deleterious for nuclear entry. One possible explanation is that the hyper-stable core masks viral nuclear localization signals of matrix, integrase, or Vpr [30–32], or masks a viral DNA structure, the central DNA flap, which is known to be important for nuclear targeting [33–36]. Another possibility is that host factors that are required for HIV-1 to enter the nucleus, such as importin α/importin β heterodimer [37–39], importin 7 [37,40,41], NUP153 [42], and TNPO3 [20], are unable to access the viral particles at the proper time or place. Although TNPO3 has been shown to bind HIV-1 integrase, Krishnan et al. recently showed that CA is the viral factor that dictates TNPO3 dependency [43]. Thus it is also possible that mutations in CA of NL-4/5S6/7SvifS affected the interaction between CA and TNPO3.

It is possible that the core of each HIV-1 CA mutant has its own optimal uncoating kinetics for RT production. For example, a virus with Q63/67A mutations in CA previously has been shown to uncoat more slowly than WT, but could synthesize cDNA at a level comparable to that of WT during single-round infection [4,11,12,44]. In the case of NL-4/5S6/7SvifS, the slow uncoating may be optimal for its RT, since RT production by this virus was faster than that by WT, even though the slower uncoating might be deleterious for nuclear entry. If so, it is reasonable to assume that the G-to-E substitution at the 116^th^ position of CA that reduced the core stability of NL-4/5S6/7SvifS resulted in impaired RT. Further studies, including evaluation of physical core stability and more precise analysis of RT products, are necessary to substantiate this hypothesis.

It is known that drug-resistant HIV-1 often acquires mutations that have a negative effect on viral replicative capability [45–50]. In addition, some of the resistant viruses acquire secondary mutations that do not compensate directly for the negative effects caused by the primary mutations, but instead improve another step, resulting in better replicative capability [51,52]. Similarly, the G-to-E substitution at the 116^th^ position of CA may impair RT production but compensate for a defect of NL-4/5S6/7SvifS in a subsequent step. In the present study, however, we failed to resolve the step at which the G116E substitution of CA compensates for a defect of NL-4/5S6/7SvifS, since no significant improvement was observed in the levels of 2-LTR circles (nuclear entry) nor HIV-Alu (integration) of NL-4/5SG116E6/7S at 12 hr after infection (Figure 3). The addition of the G116E mutation to NL-4/5S6/7SvifS may change the affinity of viral core for certain host factors and subsequently allow viral cDNA to be integrated at chromosome positions that are preferable for subsequent transcription. Alternatively, we might have failed (in Figure 3) to detect very small recoveries of 2-LTR circles and/or HIV-Alu levels, although these recoveries were sufficient to be detected after amplification by viral transcription (in Figure 2). Further studies would be required to elucidate the precise mechanisms by which the G116E mutation at least partially restored the impaired infectivity of NL-4/5S6/7SvifS.

We note that the uncoating process was completed within 4hr after infection (as shown in Figure 5), while the levels of the late-RT products continued to increase through 8-12 hr (as shown in Figures 3 and 4). Similar delay in accumulation of late-RT product compared with uncoating kinetics was reported previously [44]. Since a fluorescence-labeled antibody was used to detect assembled CAs of the pre-uncoating cores in the uncoating assay, it is likely that some cores undergoing uncoating became undetectable in this assay but still continued RT production. At present, the precise role of CA in nuclear entry and integration of HIV-1 remains to be elucidated. Further studies would be needed to determine the number of CA molecules required for efficient nuclear entry and integration of HIV-1 pre-integration complex.

It should be noted here that the amounts of p24 from culture supernatants of 293T cells transfected with NL-4/5S6/7SvifS and NL-4/5SG116E6/7SvifS plasmid constructs were approximately 75% of those of NL-vifS (data not shown). These results suggested that the viral assembly step also is impaired in NL-4/5S6/7SvifS, and that the G-to-E substitution in NL-4/5SG116E6/7SvifS fails to compensate for the mild defect in assembly of NL-4/5S6/7SvifS. Therefore, defects in both early and late viral replication steps may contribute to the impaired replicative capabilities of NL-4/5S6/7SvifS and NL-4/5SG116E6/7SvifS in human cells. It is also possible that NL-4/5S6/7SvifS has defects in steps other than those assessed in the present study.

In the study presented here, we showed that a simian-tropic HIV-1, NL-4/5S6/7SvifS, exhibited both slower uncoating and a defect in nuclear entry. On the other hand, the adapted virus NL-4/5SG116E6/7SvifS showed recovered uncoating kinetics. In addition to the Q63/67A mutant, 4/5S6/7S is the second example showing the association of slower uncoating with a disadvantage in nuclear entry. However, it is too early to generalize from this conclusion, and further studies on various other CA mutants would be required to elucidate the precise role of uncoating kinetics in HIV-1 replication.

## Conclusions

Our results suggest that the lower replicative capability of NL-4/5S6/7SvifS in human cells is due to the slower uncoating of this virus.

## Supporting Information

Table S1 **Actual numbers of dots in uncoating assay.**
(DOCX)
