# Supplementary material for: Slower Uncoating Is Associated with Impaired Replicative Capability of Simian-Tropic HIV-1
Source: PLoS One. 2013 Aug 13;8(8):e72531. doi: 10.1371/journal.pone.0072531 (PMC3742594; doi:10.1371/journal.pone.0072531)
Supplement: Table S1 — Actual numbers of dots in uncoating assay. (DOCX) [file pone.0072531.s001.docx]

Table S1. Actual numbers of dots in uncoating assay.

| Viral capsid | Time point |  | Formula | Exp. 1 | Exp. 2 | Exp. 3 |
| --- | --- | --- | --- | --- | --- | --- |
| WT | 0hr |  | GFP+p24+/GFP+ | 49/66 | 74/101 | 73/84 |
|  | 1hr |  | GFP+S15-p24+/GFP+S15- | 28/84 | 31/98 | 91/213 |
|  | 2hr |  | GFP+S15-p24+/GFP+S15- | 30/96 | 53/204 | 63/179 |
|  | 4hr |  | GFP+S15-p24+/GFP+S15- | 22/68 | 42/127 | 16/57 |
|  | 4hr+Baf |  | GFP+p24+/GFP+ | 123/143 | 92/123 | 123/162 |
|  |  |  |  |  |  |  |
| 4/5S6/7S | 0hr |  | GFP+p24+/GFP+ | 47/62 | 66/89 | 77/96 |
|  | 1hr |  | GFP+S15-p24+/GFP+S15- | 41/72 | 67/134 | 142/286 |
|  | 2hr |  | GFP+S15-p24+/GFP+S15- | 50/114 | 147/352 | 158/393 |
|  | 4hr |  | GFP+S15-p24+/GFP+S15- | 45/112 | 49/126 | 27/85 |
|  | 4hr+Baf |  | GFP+p24+/GFP+ | 96/117 | 92/100 | 117/167 |
|  |  |  |  |  |  |  |
| 4/5S6/7SG116E | 0hr |  | GFP+p24+/GFP+ | 47/70 | 68/94 | 70/82 |
|  | 1hr |  | GFP+S15-p24+/GFP+S15- | 54/123 | 38/97 | 118/276 |
|  | 2hr |  | GFP+S15-p24+/GFP+S15- | 49/125 | 84/235 | 127/339 |
|  | 4hr |  | GFP+S15-p24+/GFP+S15- | 58/134 | 50/151 | 28/99 |
|  | 4hr+Baf |  | GFP+p24+/GFP+ | 112/137 | 99/121 | 194/290 |

WT: wild type
